# Prophylactic Role of Ketamine and Dexmedetomidine on the Prevention of Shivering in Patients Undergoing Inguinal Herniorrhaphy by Spinal Anesthesia: A Randomized Clinical Trial

**DOI:** 10.5812/aapm-145266

**Published:** 2025-12-16

**Authors:** Vahid Adiban, Masood Entezariasl, Pedram Poshtareh, Khatereh Isazadehfar

**Affiliations:** 1 Department of Anesthesiology, Ardabil University of Medical Sciences, Ardabil, Iran; 2 Department of Anesthesiology, Ilam University of Medical Sciences, Ilam, Iran; 3 Department of Community Medicine, Social Determinants of Health Research Center, School of Medicine, Ardabil University of Medical Sciences, Ardabil, Iran

**Keywords:** Dexmedetomidine, Herniorrhaphy, Ketamine, Shivering

## Abstract

**Background:**

Numerous medical interventions have been utilized to prevent postoperative shivering. Due to the potential complications associated with the use of pethidine, such as respiratory failure, the exploration of alternative drugs for the prevention and treatment of postoperative shivering has been a key consideration.

**Objectives:**

The aim of this study was to assess the preventive effects of ketamine and dexmedetomidine on shivering in patients undergoing inguinal herniorrhaphy under spinal anesthesia.

**Methods:**

This triple-blind randomized clinical trial involved patients who were candidates for inguinal herniorrhaphy with spinal anesthesia. Patient, investigator/administering, and outcome assessor were blinded. The necessary sample size was 150, estimated based on statistical formula at a 95% confidence interval and 80% power. Patients were randomly assigned using a computer-generated random sequence allocation and a randomized block sampling design with block sizes of six to ensure balanced allocation across the three groups: Ketamine, dexmedetomidine, and control. Randomization was performed using random sequence allocation software and randomized block sampling with 6 blocks for all 3 treatment groups. The severity of shivering was assessed using the Bedside Shivering Assessment Scale (BSAS) at multiple time points: Immediately, 5, 15, and 30 minutes after spinal anesthesia, and upon entering the recovery room, 15 minutes later, and at discharge from recovery. Data were analyzed using IBM SPSS Statistics version 21 software. Quantitative data were expressed as mean ± standard deviation, while qualitative data were presented as percentages. The mean of the variables was compared using Student's *t*-test, and the chi-square test was employed to compare qualitative data.

**Results:**

The severity of shivering was notably lower in the dexmedetomidine group at 5, 15, and 30 minutes after spinal anesthesia induction, during recovery, and 15 minutes after recovery. The intensity of shivering upon exiting recovery was similar in the ketamine and dexmedetomidine groups and significantly lower than in the control group. Systolic blood pressure was significantly lower in the dexmedetomidine group upon entry into recovery and 15 minutes after recovery. Throughout all time periods, patients in the dexmedetomidine group exhibited significantly lower heart rates.

**Conclusions:**

Both ketamine and dexmedetomidine proved effective in reducing post-herniorrhaphy shivering compared to the control group, with the effect being notably greater in the dexmedetomidine group.

## 1. Background

Inguinal hernia is a protrusion of the contents of the abdomen through the inguinal canal and is very common. Inguinal herniorrhaphy is one of the most frequently performed surgeries by general surgeons and is mainly conducted under spinal anesthesia (1, 2). Numerous drugs have been tried as adjuncts to spinal anesthesia to reduce pain and prolong postoperative analgesia; however, most of them cause significant side effects. For example, opioids are widely used to relieve postoperative pain in both spinal and epidural anesthesia. Although the duration of spinal anesthesia leads to some side effects such as nausea and vomiting, intolerance, pruritus, urinary retention, and respiratory failure, it does not lead to a delay in the patient's movement (3, 4).

Dexmedetomidine is an alpha-2 agonist with pharmacological properties that can control pain, agitation, and delirium in the intensive care unit. When administered intravenously, it provides a moderate level of relief that is easily reversible. It also reduces anxiety, pain, and sympathetic stimulation with minimal effect on respiratory function (5). There is much evidence of beneficial and protective effects of this drug on heart, kidney, and brain function in patients (6).

Ketamine is an N-methyl-D-aspartate (NMDA) receptor antagonist and is part of the anesthetic drug class. Administration of a low dose of ketamine before induction of anesthesia prevents severe hemodynamic changes while providing appropriate analgesia (7). It appears that the NMDA receptor has a role in transmitting temperature neural messages to the brain and spinal cord. Ketamine has an antagonistic effect on the NMDA receptor and is also a cheap and available compound in anesthesia. It differs from other analgesic compounds in that it has pronounced analgesic effects and does not counteract respiratory and cardiovascular depression (8).

Shivering is an involuntary movement of muscle that occurs physiologically in response to hypothermia. Prolonged disturbance of the temperature autonomic control center, the cold environment of the operating room, and cold prescribed fluids cause a drop in body temperature and consequently shivering (4). Shivering is common in the postoperative period and varies according to age, sex, and length of operation (9). The prevalence of postoperative shivering in spinal anesthesia has been reported in the range of 40 - 70% (10). Numerous pharmacological interventions have been proposed to treat postoperative shivering. Many opioid derivatives are widely used to treat shivering. Meperidine is a potent opioid which is also frequently used to control shivering after general anesthesia. There are some controversies about the effects of opioid derivatives to control postoperative shivering (11). Spinal anesthesia is a safe and popular method for various surgeries (12).

Regional anesthesia, due to a decrease of -5°C in the threshold of body temperature regulation, intensifies vascular contraction and causes shivering above the surface of the block. This decrease in the threshold is proportional to the age, level of sensory block, and the number of blocked segments (13).

Numerous narcotic and non-narcotic substances have been employed to prevent postoperative shivering. Certain medications, including meperidine (pethidine), have demonstrated effectiveness in treating postoperative shivering across all dosage levels, but due to the potential side effects such as respiratory weakness following the use of pethidine, finding alternative medications for the prevention and treatment of postoperative shivering has always been considered (14).

According to findings of previous studies, both ketamine and dexmedetomidine can reduce the rate of postoperative shivering in patients, and because each of these drugs has its own side effects (7, 9).

## 2. Objectives

The purpose of this study was to assess the impact of ketamine and dexmedetomidine on preventing shivering and the hemodynamic status of patients undergoing inguinal herniorrhaphy with spinal anesthesia.

## 3. Methods

This research was a triple-blind, randomized controlled clinical trial conducted at Fatemi Hospital of Ardabil in 2020. The study population included patients who were candidates for inguinal herniorrhaphy with spinal anesthesia. Written consent was obtained from all patients. Patients of American Society of Anesthesiologists (ASA) class I and II and in the age range between 15 and 70 were included in the study.

Patients with drug or food allergies, drug addiction, cancer, history of peptic ulcer, systemic infection or immunodeficiency, untreated hypothyroidism, benign prostatic hypertrophy and ureteral stenosis, seizure disorders, history of use of monoamine oxidase inhibitors, and patients who had significant bleeding during surgery were excluded.

The likelihood of reducing the occurrence of shivering in earlier research was approximately 20% lower compared to the control group, with a significance level of 0.05 and a statistical power of 0.2. As a result, a sample size of 50 in each group was determined to be necessary in order to detect a difference between the groups with a statistical power of 80%.

Randomization was performed using random sequence allocation software and randomized block sampling with 6 blocks for all 3 treatment groups. Consequently, the patients were allocated into three groups, each consisting of 50 individuals: The dexmedetomidine group received a dose of 1 µg/kg diluted in normal saline with a total syringe volume of 5 mL (administered immediately after spinal anesthesia), while the ketamine group received a dose of 0.3 mg/kg diluted in normal saline with a total volume of 5 mL (administered immediately after spinal anesthesia), and the control group received 5 ml normal saline (injection immediately after spinal anesthesia).

The three syringes had identical coloration (with colorless contents) and volume (5 mL of liquid in each syringe). The anesthesiologist in charge of monitoring and documenting the clinical indicators was unaware of the specific drug administered to the patients. In all patients, the level of anesthesia was up to T10, and all oral medications of all patients were discontinued from 8 hours before surgery.

Additionally, the method of anesthesia and the drugs used were the same for all groups, and all patients were injected from the L4 - L5 space with 25-gauge needles. The temperature of the operating room and recovery room was standardized.

Before entering the operating room, the study method was explained to the patients, and after obtaining written consent, they entered the study. To prevent hallucinations from ketamine, 1 mg of midazolam was injected intravenously to all three groups.

In this study, all patients underwent spinal anesthesia with marcaine 0.5% and no other additives. During surgery, patients underwent standard monitoring and oxygenation (4 liters per minute with a mask). The severity of shivering after spinal anesthesia was determined by a trained anesthesia resident using a score of 0 - 3 [Bedside Shivering Assessment Scale (BSAS)]: No shivering was observed when examining the masseter, neck, or chest wall (shivering grading = 0); mild: Shivering was limited to the neck and/or chest area only (shivering grading = 1); moderate: Shivering includes large movements of the upper limbs, in addition to the neck and chest (shivering grading = 2); Severe: Shivering involves significant movement of the torso, as well as the upper and lower limbs (shivering grading = 3).

The hemodynamic status of patients was charted during surgery. The patient's hemodynamic status and the presence and severity of shivering were recorded immediately, 5, 15, and 30 minutes after spinal anesthesia and at the time of entering the recovery, after 15 minutes, and when the patient left the recovery. In case of severe vomiting, 10 mg metoclopramide was prescribed. In case of shivering with an intensity of 2 or more, intravenous pethidine at a dose of 25 mg was used.

The statistical analysis was conducted using SPSS software version 24. Hemodynamic changes and shivering were assessed in comparison to baseline values using analysis of variance (ANOVA) and the Tukey post-hoc test. Quantitative data were expressed as mean ± standard deviation, while qualitative data were presented as percentages. The mean of the variables was compared using Student's *t*-test, and the chi-square test was employed to compare qualitative data. A significance level of P < 0.05 was considered statistically significant.

## 4. Results

In this study, a total of 150 patients were enrolled, comprising 116 males and 34 females, with a mean age of 43.8 ± 13.47 years. These patients were randomly allocated into three groups, each consisting of 50 patients. None of the patients were excluded during the study. The three groups exhibited similar mean age and sex distribution, and no statistically significant differences were observed between the groups (Table 1). 

**Table 1.** Comparison of Sex and Age of Patients ^a^

Group	Age		Gender
	Male	Female	
**Ketamine**	12.54 ± 42.14	36	14
**Dexmedetomidine**	13.87 ± 46.40	42	8
**Control**	13.95 ± 42.93	38	12
**P-value**	0.261	0.340	

^a^ Values are expressed as mean ± SD.

Table 2 indicates statistically significant variances in heart rate, systolic blood pressure, and diastolic blood pressure among the groups. Systolic and diastolic blood pressure status at initiation, 5, 15, and 30 minutes after spinal anesthesia were not statistically significant between the groups, but during entry into recovery, 15 minutes after recovery, and at exit from recovery, they were significantly lower in the dexmedetomidine group. The heart rate was significantly lower in the dexmedetomidine group across all measurements (Table 2). 

**Table 2.** Changes in Systolic Blood Pressure, Diastolic Blood Pressure, and Heart Rate Between Groups ^a^

Variables and Time	Group			P
	Ketamine	Dexmedetomidine	Control	
**SBP**				
Early	137.1 ± 85.4	129.2 ± 18.2	126 ± 19.4	0.53
5th minute	122 ± 15	123.2 ± 18.9	122.6 ± 14.6	0.92
15th minute	120.6 ± 14.5	117.3 ± 16	118.7 ± 11.3	0.5
30th minute	118.9 ± 11.2	113.3 ± 14.6	117 ± 11.1	0.76
Enter recovery	118.2 ± 11.3	110.8 ± 13.4	117.4 ± 12	0.005 ^b^
15 minutes after recovery	120.8 ± 12	110.7 ± 13.4	117.4 ± 10.2	< 0.0001 ^c^
Exit	119.2 ± 12.3	110.2 ± 11.6	117.1 ± 9.7	< 0.0001 ^c^
**DBP**				
Early	79.24 ± 10.8	79.9 ± 10.1	82.1 ± 13.6	0.43
5th minute	75.9 ± 8.5	74.9 ± 12	74.3 ± 9.3	0.72
15th minute	76.1 ± 6.7	72.8 ± 11.4	72 ± 9.7	0.08
30th minute	73.7 ± 7.3	69.2 ± 11.3	71.2 ± 8.9	0.06
Enter Recovery	76.2 ± 6.7	68.2 ± 12.3	72.4 ± 11.5	0.001 ^b^
15 minutes after recovery	78.9 ± 6.9	68.14 ± 11.6	71.2 ± 10.1	< 0.0001 ^c^
Exit	77.6 ± 7.4	68.1 ± 9.9	70.2 ± 10.2	< 0.0001 ^c^
**Heart rate**				
Early	79.9 ± 10.9	75.3 ± 15.4	85.3 ± 13.2	0.001 ^b^
5th minute	77.7 ± 10.5	62.8 ± 12.9	81.2 ± 12.8	< 0.0001 ^c^
15th minute	75.4 ± 10.6	60.8 ± 12.3	76.8 ± 11.4	< 0.0001 ^c^
30th minute	74.5 ± 11.1	60.6 ± 11.1	72.2 ± 15.7	< 0.0001 ^c^
Enter Recovery	75.2 ± 11.6	61.4 ± 11.7	78.312.2	< 0.0001 ^c^
15 minutes after recovery	76 ± 11	60.8 ± 10.1	79 ± 11.8	< 0.0001 ^c^
Exit	76.4 ± 9.1	61.5 ± 10.1	77.9 ± 13.3	< 0.0001 ^c^

^a^ Values are expressed as mean ± SD.

^b^ P < 0.05 was considered statistically pronounced significant.

^c^ P < 0.001 was considered statistically pronounced significant (early: Immediately after spinal anesthesia, enter: Time to enter recovery, exit: Time to exit recovery).

Although several cases of shivering were observed in the ketamine and control groups during the study period, only one case (2%) of shivering was observed 5 minutes after spinal anesthesia in the dexmedetomidine group (Table 3). 

**Table 3.** Comparison of Shivering Levels Among Groups in Different Time Periods ^a^

Time	Ketamine	Dexmedetomidine	Control	P-Value
**Early**				0.007 ^b^
Score 0	40 (80)	48 (96)	48 (96)	
Score 1	10 (20)	1 (2)	2 (4)	
Score 2	0 (0)	0 (0)	0 (0)	
Score 3	0 (0)	1 (2)	0 (0)	
**5th minute**				0.001 ^b^
Score 0	36 (75)	49 (98)	46 (92)	
Score 1	12 (25)	1 (2)	4 (8)	
Score 2	0 (0)	0 (0)	0 (0)	
Score 3	0 (0)	0 (0)	0 (0)	
**15th minute**				0.038 ^b^
Score 0	46 (92)	50 (100)	42 (84)	
Score 1	4 (8)	0 (0)	6 (12)	
Score 2	0 (0)	0 (0)	2 (4)	
Score 3	0 (0)	0 (0)	0 (0)	
**30th minute**				0.013 ^b^
Score 0	44 (88)	50 (100)	38 (76)	
Score 1	4 (8)	0 (0)	4 (8)	
Score 2	2 (4)	0 (0)	6 (12)	
Score 3	0 (0)	0 (0)	2 (4)	
**Enter Recovery**				< 0.0001 ^c^
Score 0	46 (92)	50 (100)	28 (56)	
Score 1	4 (8)	0 (0)	12 (24)	
Score 2	0 (0)	0 (0)	4 (8)	
Score 3	0 (0)	0 (0)	6 (12)	
**After 15 minutes**				< 0.0001 ^c^
Score 0	47 (94)	50 (100)	26 (54)	
Score 1	3 (6)	0 (0)	18 (37)	
Score 2	0 (0)	0 (0)	4 (8.3)	
Score 3	0 (0)	0 (0)	0 (0)	
**Exit time**				< 0.0001 ^c^
Score 0	50 (100)	50 (100)	30 (60)	
Score 1	0(0)	0(0)	20 (40)	
Score 2	0(0)	0(0)	0(0)	
Score 3	0(0)	0(0)	0(0)	

^a^ Values are expressed as No. (%).

^b^ P < 0.05 was considered statistically significant.

^c^ P < 0.001 was considered statistically pronounced significant (early: Immediately after spinal anesthesia, enter: Time to enter recovery, exit: Time to exit recovery).

At 15 and 30 minutes after spinal anesthesia, as well as upon entry into and 15 minutes after recovery, the severity of shivering was notably reduced in the ketamine group compared to the control group. No instances of shivering were observed in the ketamine and dexmedetomidine groups upon exiting recovery, while 40% of patients in the control group experienced shivering (Table 3). 

## 5. Discussion

Of the many adjuncts used in spinal anesthesia to reduce pain and prolong analgesia, most cause significant side effects. Ketamine and dexmedetomidine are two recent candidates noted for their efficacy in reducing shivering with a favorable side-effect profile.

The exact cause of shivering under spinal anesthesia is not fully understood. The sympathetic blockade below the level of the spinal block inhibits vasoconstriction in the lower body, leading to vasoconstriction and shivering primarily in the unblocked upper regions. This affects core body temperature, making central thermoregulation, a reduced shivering threshold, and environmental heat exchange significant contributing factors (15).

Ketamine may control shivering in at-risk patients by inducing sympathetic stimulation and vasoconstriction, acting on the hypothalamus, or via the alpha-adrenergic effects of norepinephrine. Dexmedetomidine reduces shivering by binding to alpha-2 receptors, causing vasoconstriction, and through its effects on hypothalamic thermoregulation (16). Its sedative properties also improve patient comfort, hemodynamic stability, and amnesia (17).

Our study included 150 patients (116 male, 34 female). The mean age was similar across groups, but a significant difference in sex distribution reflected the higher prevalence of herniorrhaphy surgery in men. Comparing heart rates after spinal anesthesia, the control group decreased from 85 to 81 bpm, the dexmedetomidine group from 75 to 62 bpm, and the ketamine group from 79 to 77 bpm. The more pronounced decrease in the dexmedetomidine group highlights the influence of measurement timing.

A study by McVey and Tobias (18) found that co-administration of dexmedetomidine and ketamine increased heart rate and systolic blood pressure but provided favorable sedation with no adverse respiratory effects. Conversely, Alvarez Corredor (19) found meperidine most effective at reducing shivering, with no significant difference between dexmedetomidine and ketamine. In our study, both drugs significantly reduced shivering compared to the control, but dexmedetomidine was statistically superior to ketamine.

Ameta et al. (20) also found dexmedetomidine superior to ketamine and tramadol for shivering control, though with a higher incidence of hypotension. Our study found no such hypotensive episodes requiring intervention. Koruk et al. (21) reported a higher heart rate with ketamine compared to dexmedetomidine in children, with no difference in shivering. Finally, a study by Ghasemi et al. (15) in addicted patients found dexmedetomidine reduced shivering without adverse hemodynamic effects, aligning with our results.

### 5.1. Conclusions

This triple-blind, randomized controlled trial demonstrates that prophylactic intravenous dexmedetomidine (1 µg/kg) and ketamine (0.3 mg/kg) are both effective in reducing shivering following spinal anesthesia for herniorrhaphy compared to placebo, with dexmedetomidine proving significantly more effective. This finding is consistent with the existing literature, where dexmedetomidine's potent central alpha-2 adrenergic agonist action is well-established as a powerful anti-shivering mechanism.

However, a comprehensive conclusion must also address limitations and potential harms. While dexmedetomidine provided superior hemodynamic stability, this effect is a double-edged sword; its significant sympatholytic properties can lead to clinically important bradycardia and hypotension, adverse events that were not quantified in this report but are well-documented in other studies. Conversely, while ketamine was less effective for shivering, its tendency to cause hypertension, tachycardia, or psychomimetic emergence reactions (mitigated here by midazolam premedication) represents its own distinct risk profile. When compared with other studies, our results align with the consensus on dexmedetomidine's efficacy but may contrast with research suggesting ketamine's effect is more potent, a discrepancy potentially explained by differences in dosing, patient population, or ambient temperature control.

In summary, while dexmedetomidine is the most effective anti-shivering agent in this study, its choice over ketamine requires a careful, patient-specific risk-benefit analysis weighing the superior shivering reduction and stability against the potential for bradycardia. Future research should directly report the incidence of these specific adverse events to provide a clearer comparative safety profile.

### 5.2. Limitations

The limitation of this study was that it was not performed on long-term surgeries (the average duration of surgery in both groups was approximately half an hour), as long-term surgeries could cause hypothermia in patients. In addition, different doses of dexmedetomidine have not been studied in this research.

## Data Availability

The primary database for this study is available upon request to the corresponding author.

## Clinical Trial Registration Code:

This trial was registered at the Iran Trial Registration Center (IRCT20190930044937N1).
